# ‘HELPING ALLEVIATE LONELINESS IN HONG KONG OLDER ADULTS’ (HEAL-HOA): A DUAL RANDOMIZED CONTROLLED TRIAL

**DOI:** 10.1093/geroni/igae098.1068

**Published:** 2024-12-31

**Authors:** Da Jiang, Jojo Kwok, Namkee Choi, Dannii Y Yeung, Rainbow Tin Hung Ho, Lisa Warner, Kee-Lee Chou

**Affiliations:** Education University of Hong Kong, Hong Kong, China (People’s Republic); The University of Hong Kong, Hong Kong, China (People’s Republic); The University of Texas Austin, Austin, Texas, United States; City University of Hong Kong, Hong Kong, China (People’s Republic); The University of Hong Kong, Hong Kong, China (People’s Republic); MSB Medical School Berlin, Berlin, Germany; Education University of Hong Kong, Hong Kong, China (People’s Republic)

## Abstract

Despite the high prevalence and negative impact of loneliness, research on the effectiveness of behavioral interventions for older adults who are experiencing loneliness has been lacking. The ‘Helping Alleviate Loneliness in Hong Kong Older Adults’ (HEAL-HOA), a dual randomized controlled trial (RCT), tested the effects of telephone-based psychosocial interventions delivered by older-adult volunteers (N=375) for lonely older adults (N=1,151) during the COVID-19 pandemic. The 375 volunteers age 50-70 were randomized into a volunteering condition versus an active control. Those in the volunteering condition received six 2-hour training on behavioral activation (Tele-BA), mindfulness (Tele-MF), or befriending (Tele-BF). Those in the active control condition received psychoeducation on loneliness. Trained volunteers then delivered Tele-BA, Tele-MF, or Tele-BF (active control) as lay counsellors for low-income, lonely, digitally excluded older adults (Mage=76.6, SDage=7.8) who lived alone and were randomized to one of three conditions. Outcomes were assessed at baseline and 6 months post-intervention among volunteer lay counsellors, and at baseline and 4 weeks, 3 months, and 6 months post-intervention among intervention recipients. For volunteer-counsellors, positive effects were observed in loneliness, social network engagement, stress and depressive symptoms as compared to those in the control condition. For intervention recipients, compared to Tele-BF, Tele-BA demonstrated positive effects on loneliness, psychological well-being, perceived support, and sleep quality at 3-month and 6-month follow-ups. Compared to Tele-BF, Tele-MF was effective in reducing loneliness at the 3-month follow-up. This innovative dual RCT demonstrated feasibility of delivering large-scale telephone-based behavioral interventions for older adults by older-adult volunteers and the intervention effectiveness.

